# Evaluation of the Antihyperglycemic and Antihyperlipidemic Activity of *Saussurea hypoleuca* Root in Alloxan-Induced Diabetes in Rat Model and Correlation to Its Major Secondary Metabolites

**DOI:** 10.3390/life12091451

**Published:** 2022-09-19

**Authors:** Numera Arshad, Saiqa Ishtiaq, Sairah Hafeez Kamran, Muhammad Sajid-ur Rehman, Shehla Akbar, Saira Rehman, Sarah Rehman, Rawan H. Hareeri, Sana A. Fadil, Fadia S. Youssef, Sameh S. Elhady

**Affiliations:** 1 Department of Pharmacy, College of Pharmacy, University of the Punjab, Lahore 05422, Pakistan; 2 The Sahara College of Pharmacy & Allied Health Sciences, Punjab 05422, Pakistan; 3 Institute of Pharmacy, Faculty of Pharmaceutical and Allied Health Sciences, Lahore College for Women University, Lahore 05422, Pakistan; 4 Department of Pharmacology and Toxicology, Faculty of Pharmacy, King Abdulaziz University, Jeddah 21589, Saudi Arabia; 5 Department of Natural Products, Faculty of Pharmacy, King Abdulaziz University, Jeddah 21589, Saudi Arabia; 6 Department of Pharmacognosy, Faculty of Pharmacy, Ain-Shams University, Abbasia, Cairo 11566, Egypt

**Keywords:** *Saussurea hypoleuca*, antihyperglycemic, antihyperlipidemic, molecular modeling, industrial development, drug discovery

## Abstract

*Saussurea hypoleuca* belongs to the family Asteraceae, which has previously shown hepatoprotective, anticancer, and antioxidant activity. This study aimed to evaluate the antihyperglycemic and antihyperlipidemic activity of its root methanol extract and various fractions for the first time. This was performed using alloxan-induced diabetes in the rat model for both short, and long-term periods using different administration doses. Different biochemical parameters were studied and further consolidated by histopathological examination and in silico molecular modeling. The results showed that in the long-term study, at a dose of 400 mg/kg b.wt, the ethyl acetate fraction caused a pronounced reduction in fasting blood glucose level (FBG) and glycated hemoglobin (HbA_1c_) by 77.2% and 36.8%, respectively, compared to the diabetic group. This was confirmed by the histopathological examination of the animals’ pancreatic sections. The ethyl acetate fraction also showed a reduction in total cholesterol (TC), total glycerides (TG), and low-density lipoprotein cholesterol (LDL-C) levels. It improved kidney and liver functions, causing a reduction in aspartate aminotransferase (AST), alkaline phosphatase (ALP), alanine transaminase (ALT), urea, and creatinine levels. This is mainly attributed to its richness in secondary metabolites. Molecular docking showed that all the tested compounds showed certain inhibitory potential towards human α-glucosidase (HAG) and ATP citrate lyase (ACL). Thus, *Saussurea hypoleuca* roots can help in the management of hyperglycemia, hyperlipidemia, and hepatic and kidney dysfunction.

## 1. Introduction

Diabetes mellitus is a metabolic disorder characterized by abnormal glucose and lipid metabolism and accompanied by characteristic long-term complications, including retinopathy, nephropathy, and neuropathy [1]. Hyperglycemia and hyperlipidemia are mutual characteristics of diabetes mellitus that stimulate the progression of micro- and macrovascular complications, leading to an increased mortality and morbidity rate [2,3,4].

Thus, searching for natural compounds that control hyperglycemia, and hyperlipidemia to ameliorate oxidative stress can be useful in inhibiting diabetes-associated complications [5,6]. Numerous drugs are available for controlling diabetes, but they also show numerous adverse effects and are expensive. Therefore, there is a great need to explore safer, economical, and innovative natural compounds with higher acceptability than synthetic entities and that are welcomed by a large category of patients [7,8,9].

The family Asteraceae includes about 1000 genera, commonly distributed in European and Asian areas. The genus *Saussurea* constitutes the largest subgenus of this family, comprising about 400 species that are distributed across the globe [10]. Traditionally, members of the genus *Saussurea* were employed to alleviate fever and pain, harmonize menstruation, increase energy production, and stop bleeding [11]. In addition, numerous species of *Saussurea* were used for the treatment of ulcers, dysentery, rheumatic arthritis, dysmenorrhea and stomach aches and as an anti-inflammatory, analgesic, cardiotonic, abortifacient, anticancer, and antifungal agent in Chinese medicinal systems [12]. It is commonly known for its richness in lignans, flavonoids, steroids, and sesquiterpene lactones, to which many of its biological activities are attributed. These biological activities include anticancer, anti-inflammatory, antioxidant, and immunomodulatory potential [10].

*Saussurea hypoleuca* Spreng. ex DC., also known as Qust, exists in Quetta in the Pakistan Mountains and the Himalayan region of India [13]. Previous studies by the authors on *S. hypoleuca* revealed the richness of the roots with phenolic acids, such as sinapic and caffeic acids, in addition to flavonoids, such as myricetin, luteolin, kaempferol, and quercetin. In addition, oleic acid, hexadecanoic acid, decanedioic acid, isopropyl myristate, tetracosapentaene and tetracosapentaene, 3,4-hexanedione were detected in the roots [14,15,16]. Additionally, the authors previously investigated the hepatoprotective, anticancer, antioxidant, anti-inflammatory and anthelmintic activity of the root extracts [17].

In continuation to the previous studies on *S. hypoleuca* roots, herein, the antihyperglycemic and the antihyperlipidemic activity of its total methanol extract was investigated in alloxan-induced diabetic rats using both short- and long-term studies for the first time. The assessment of several biochemical parameters, consolidated by histopathological examination, was also performed. A molecular modeling study was conducted on previously identified compounds detected by the authors in the roots to assess their inhibitory potential versus human α-glucosidase and ATP citrate lyase to interpret their possible mode of action using Discovery Studio 4.5 (Accelrys Inc., San Diego, CA, USA).

## 2. Materials and Methods

### 2.1. Plant Material

The roots of *Saussurea hypoleuca* Spreng. ex DC (Asteraceae) were collected from the mountains of Quetta, Baluchistan, Pakistan in September 2016 and authenticated by Prof Dr. Zaheer-ul-deen, Department of Botany, Government College University Lahore, Pakistan. The specimen was kept under the voucher number (GC. Herb. Bot. 3453) in the Government College University Herbarium Museum, Lahore, Pakistan.

### 2.2. Preparation of the Plant Extract

Air-dried roots (7 kg) were pulverized and macerated in methanol (3 × 7 L) for seven days for the preparation of extract by cold maceration. The extract was filtered and dried under reduced pressure at 45–50 °C. The methanol extract (500 g) was solubilized in water and filtered. Then, the extract was fractionated with various solvents of increasing polarity, namely *n*-hexane, chloroform, ethyl acetate, and *n*-butanol. All the fractions were evaporated until dry to yield organic fractions, which were *n*-hexane (25 g), chloroform (30 g), ethyl acetate (55 g), *n*-butanol (80 g), and the remaining aqueous fraction (295 g) that was subsequently preserved at 4 °C prior to use [18].

### 2.3. Chemicals and Reagents

All chemicals and reagents used in this study were of analytical grades and purchased from Sigma Chemical Co., St Louis, MO, USA.

### 2.4. In Vivo Antihyperglycemic and Antihyperlipidemic Evaluation

#### 2.4.1. Animals and Animal Treatment

Male and female Albino Wistar rats (150–200 g) were obtained from the Department of Pharmacology, University College of Pharmacy, Punjab University, Lahore, Pakistan. They were kept in metal cages at controlled room temperature at 25 ± 2 °C, with free access to food and water ad libitum for one week before the beginning of the experiment. Approval for the experimental protocol was obtained from the institutional committee for animals handling and care under voucher no.88.

#### 2.4.2. Acute Oral Toxicity Study

Before performing the in-vivo biological studies, the extract and its fractions were tested for acute oral toxicity using OECD guidelines (421 and 422). The extract and its fractions at 200 mg/kg, 400 mg/kg, 1 g/kg, and 2 g/kg were given to mice for 14 days, and then, signs of toxicity were observed [19].

#### 2.4.3. Induction of Diabetes

Diabetes was induced by intraperitoneal administration of a freshly prepared aqueous solution of alloxan monohydrate (150 mg/kg b.wt) after overnight fasting in all groups, except the normal group. Animals were given 5% glucose solution for the next 24 h instead of simple water. Blood glucose levels were tested after 72 h of alloxan administration. Animals with fasting glucose levels of more than 200 mg/dL were considered diabetic [20].

#### 2.4.4. Experimental Protocol

##### Short-Term Study

Animals were allocated into eight groups, with six rats in each group. In the first group, the normal group, normal animals orally received distilled water only. In contrast, the second group was the diabetic control group, where the diabetic animals received no treatment but distilled water only. Meanwhile, from the third to the eighth groups, diabetic rats were treated with methanol, *n*-hexane, chloroform, ethyl acetate, *n*-butanol, and the remaining aqueous fractions in a dose of 200 mg/kg b.wt in 1 mL of distilled water, respectively, after overnight fasting. The measured dose of each tested sample was suspended in water and vortexed for 1 min to ensure its homogenous suspension in water, before being administered orally through oral gavage. Blood was collected from the tail vein at 0, 1, 2, 3, 4, 5, and 6 h after each extract administration, and glucose was determined by oxidase-peroxidase reactive strips (Accu-chek, Roche Diagnostics, GmbH, Mannheim, Germany) [21].

##### Different Administration Doses Study

Animals were allocated into seven groups; each group consisted of six rats. In the first group, the normal group, normal animals orally received distilled water only. The second group was the diabetic control group, where diabetic animals received no treatment but distilled water. Meanwhile, in the third and fourth groups, diabetic rats received 200 and 400 mg/kg b.wt of ethyl acetate fraction in 1 mL distilled water, whereas in the fifth and sixth groups, diabetic rats received 200 and 400 mg/kg b.wt of the root methanol extract in 1 mL distilled water. In the seventh group, diabetic rats were treated with 10 mg/kg b.wt of glibenclamide in 1 mL of distilled water. The measured doses of each tested sample were suspended in water and vortexed for 1 min to ensure its homogenous suspension in water, before being administered orally through oral gavage [22]. After overnight fasting, blood was collected from the tail vein at 0, 1, 2, 3, 4, 5, and 6 h to determine blood glucose levels using oxidase-peroxidase reactive strips [23].

##### Long-Term Study

In the long-term study, animals were allocated into five groups, each with six rats. In the first group (the normal group), normal animals orally received distilled water only. The second group was the diabetic control group, in which the diabetic animals received no treatment but distilled water. Meanwhile, in the third and fourth groups, diabetic rats received 400 mg/kg b.wt of the methanol extract and ethyl acetate fraction in 1 mL distilled water for 30 days. In the fifth group, the diabetic rats were treated with 10 mg/kg b.wt of glibenclamide in 1 mL of distilled water for 30 days. The measured dose of each tested sample was suspended in water and vortexed for 1 min to ensure its homogenous suspension in water, before being administered orally through oral gavage. Blood samples were collected from the tail vein on the first, 10th, 20th, and 30th days to determine the fasting glucose levels, Hb, HbA_Ic_, and body weights. Body weights of all animals were recorded before treatment as described by Nabi et al. [24]; meanwhile, Hb and HbA_Ic_ were estimated as reported by Eross et al. [25].

#### 2.4.5. Analytical Procedure

After overnight fasting on the 30th day, rats were sacrificed under light ether anesthesia. Blood samples were taken by cardiac puncture for biochemical analysis. Blood samples were collected in gel tubes to be centrifuged immediately at 3000 rpm for 15 min to separate the serum. The lipid profile was determined using standard kits [25,26,27,28]. The liver enzymes were determined in the blood samples after centrifugation using standard diagnostic Crescent kits [29,30,31]. Serum creatinine and urea were also determined by Jaffe’s and diacetyl monooxime methods, respectively [31,32,33].

#### 2.4.6. Histopathological Examination

The pancreases from each group of rats were preserved in 10% formalin for histopathological examination. Pancreatic tissues were dehydrated, cleared, and embedded into paraffin blocks, after being cut and stained with hematoxylin dye for histopathological documentation of the slides [34].

### 2.5. Statistical Analysis

All results were expressed as mean ± SD. Statistical analysis was performed using the Student’s t-test and one-way analysis (ANOVA), followed by Duncan’s multiple range test (DMRT). Graphs were constructed using GraphPad Prism version 5 software (GraphPad Software, Inc. La Jolla, CA, USA)

### 2.6. In Silico Molecular Docking Study

The molecular modeling study was conducted on the identified compounds previously detected in the roots by the authors [14,15,16,17] to assess their inhibitory potential versus human α-glucosidase HG) (PDB ID 3TOP; 2.88 Å) and ATP citrate lyase (ACL) (PDB ID 3MWD; 2.10 Å) obtained from the protein data bank (www.pdb.org, accessed on 16 June 2022). This was performed to predict their probable mode of action in alleviating hyperglycemia and hyperlipidemia and to interpret their possible mode of action using Discovery Studio 4.5 (Accelrys Inc., San Diego, CA, USA) via C-docker protocol, following what was previously reported [35,36,37]. 

## 3. Results and Discussion

### 3.1. Functional Substances Predominating in Saussurea hypoleuca Roots

**Figure 1:**
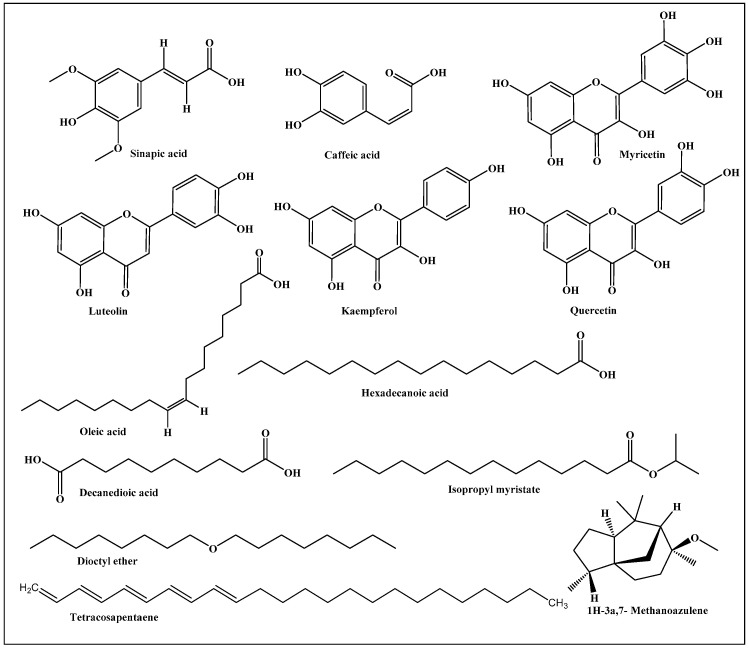
Scheme showing previously identified major compounds prevailing in *Saussurea hypoleuca* root.

*Saussurea hypoleuca* revealed the richness of the roots with phenolic acids, such as sinapic and caffeic acids, in addition to flavonoids such as myricetin, luteolin, kaempferol, and quercetin. In addition, oleic acid, hexadecanoic acid, decanedioic acid, isopropyl myristate, tetracosapentaene, and dioctyl ether and 1H-3a,7-methanoazulene were detected in the roots [14,15,16]. A scheme that shows the previously identified major compounds prevailing in the root is presented in Figure 1.

### 3.2. Acute Oral Toxicity Study

In the oral acute toxicity study, the extract and its fractions at 200 mg/kg, 400 mg/kg, 1 g/kg, and 2 g/kg were given to mice for 14 days. No sign of toxicity was observed. Therefore, it was conceivable that the plant extract, and its fractions were safe up to 1 g/kg. Based on the acute toxicity testing, two doses of 200 and 400 mg/kg were selected for the following biological studies.

### 3.3. In Vivo Antihyperglycemic Evaluation of Saussurea hypoleuca Roots

The current study was designed to evaluate the ability of *S. hypoleuca* root to treat hyperglycemia and hyperlipidemia in alloxan-induced diabetic rats. Alloxan was used for the induction of diabetes experimentally, owing to its selective damage to the pancreatic β- cells in the islets of Langerhans that produce insulin. Alloxan caused diabetes in a multiphasic pattern, causing an initial alteration in blood glucose response after being injected in an experimental rat, which was consequently followed by inverse changes in the plasma insulin, resulting in sequential ultrastructural β- cells variation that ends with ultimate necrotic cell death [38,39].

#### 3.3.1. Short-Term Study

After confirmation of the occurrence of diabetes, a short-term study was designed with the tested extract and fractions. The results illustrated in Table 1 showed that among all the tested samples of *S. hypoleuca* roots, only methanol extract and ethyl acetate fraction at a dose of 200 mg/kg b.wt produced significant effects as compared to the other tested fractions, which did not show any significant effects in diabetic treated rats. After 6 h, the FBG level was drastically elevated in diabetic rats by 360.65%, compared to the normal control group. Meanwhile, after 6 h of methanol extract and ethyl acetate fraction administration at 200 mg/kg b.wt, the FBG levels were significantly reduced by 42.7% and 77.8%, respectively, compared to the diabetic group. The ethyl acetate fraction showed a pronounced amelioration, with the FBG level approaching that of the normal control animals.

#### 3.3.2. Different Administration Dose Study

Based on the short-term study, different doses of the root methanol extract and ethyl acetate fraction at 200 and 400 mg/kg b.wt were evaluated. After 6 h of treatment, the ethyl acetate fraction reduced fasting blood glucose levels by 75% and 77%, respectively. Meanwhile, the root methanol extract produced 32% and 73% blood glucose lowering effects at 200 and 400 mg/kg b.wt, respectively, compared to the diabetic group. It is important to highlight that glibenclamide at a dose of 10 mg/kg b.wt resulted in a 30% decrease in blood glucose levels after 5 h treatment, as summarized in Table 2.

#### 3.3.3. Long-Term Study

The root methanol extract and ethyl acetate fraction at a dose of 400 mg/kg b.wt were selected for a long-term study for 30 days. The results illustrated in Table 3 showed that alloxan caused a marked elevation in FBG, estimated at 430% compared to the normal group. In contrast, treatment with the root methanol extract and ethyl acetate fraction at a dose of 400 mg/kg b.wt caused a pronounced reduction in FBG by 77.01% and 77.2% (*p* < 0.001), respectively, compared to the untreated group. They showed superior activity when compared to glibenclamide, which showed a 53.34% reduction in FBG level compared to the diabetic animals. In addition, diabetic animals exhibited a notable reduction in body weight, estimated at 21.78% compared to the normal group. On the contrary, animals treated with the root methanol extract, ethyl acetate fraction, and glibenclamide showed nearly similar body weights when compared to the normal group. It is important to mention that the body weights of all groups increased except for the animals of diabetic group, which decreased due to the loss of muscle and waste of tissue protein induced by alloxan [40] (Table 3).

**Figure 2:**
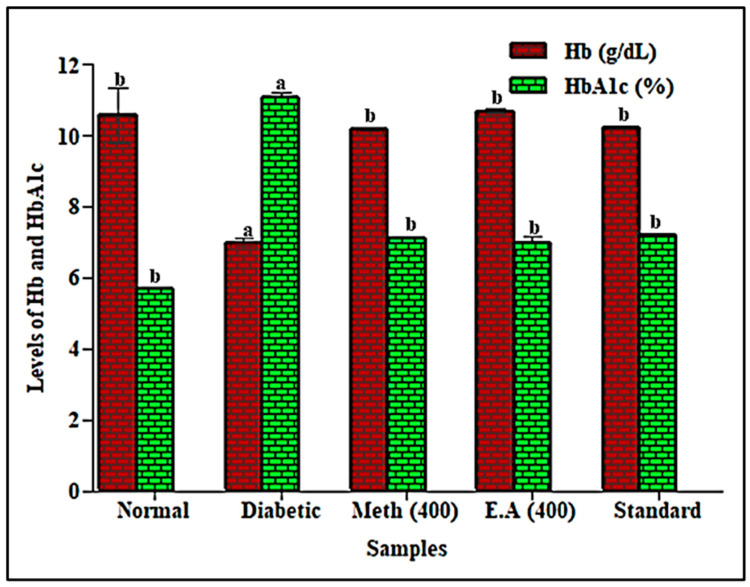
Effects of root methanol extract and ethyl acetate fraction of *S. hypoleuca* on hemoglobin (Hb) and glycated hemoglobin (HBA1c). Values are expressed as mean ± SD, *n* = 6, Hb; hemoglobin; HBA1c, glycated hemoglobin; all values that have common superscript letters differ significantly at *p* < 0.01 (DMRT); Meth (400); *S. hypoleuca* root methanol extract at a dose of 400 mg/kg b.wt,; E.A (400); *S. hypoleuca* root ethyl acetate fraction at a dose of 400 mg/kg b.wt; standard, glibenclamide at a dose of 10 mg/kg b.wt; DMRT = Duncan’s multiple range test.

HbA_1c_ is an important tool for estimating the degree of protein glycation in diabetes, where elevated blood glucose reacts with hemoglobin to generate HbA_1c_ [41]. The hemoglobin level decreased with an increased level of HbA_1c_ in diabetic rats, where HbA_1c_ was significantly elevated in diabetic rats by 95.5% compared to the normal group. In contrast, HbA_1c_ becomes stabilized in diabetic rats treated with methanol extract and ethyl acetate fraction, due to the substantial improvement in insulin secretion. The animals treated with the root methanol extract and ethyl acetate fraction exhibited a pronounced reduction in HbA_1c_, estimated at 35.7% and 36.8%, respectively, compared to the diabetic group. They showed comparable values with glibenclamide, revealing a 34.9% reduction in HbA_1c_ relative to the diabetic group (Figure 2).

### 3.4. In Vivo Antihyperlipidemic Evaluation of Saussurea hypoleuca Roots

**Figure 3:**
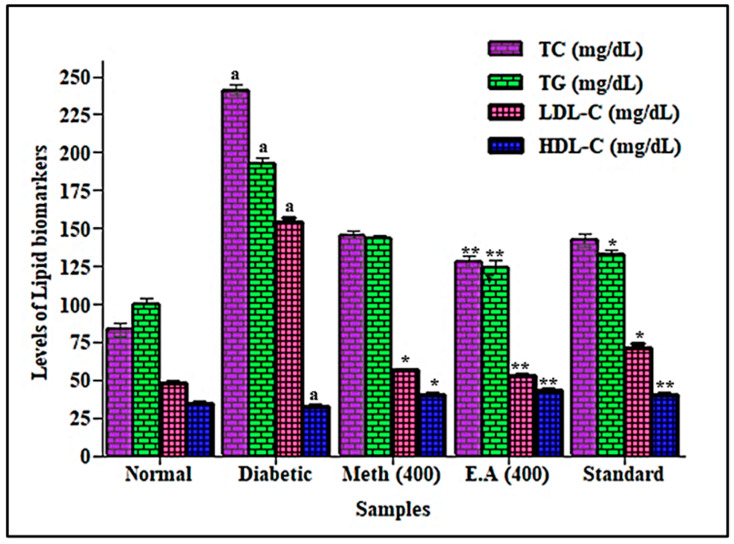
Effects of root methanol extract and ethyl acetate fraction of *S. hypoleuca* on total cholesterol (TC), total glycerides (TG), low-density lipoprotein-cholesterol (LDL-C) and high-density lipoprotein-cholesterol (HDL-C). Values are expressed as mean ± SD, *n* = 6. Data analysis was performed by Student’s *t*-test followed by one-way ANOVA. ** *p* < 0.001 and * *p* < 0.01 methanol, E.A and standard compared with diabetic rats. ^a^ comparison of diabetic rats with normal control group. TC = total cholesterol, TG = total glycerides, HDL-C = high-density lipoprotein-cholesterol and LDL-C = low-density lipoprotein-cholesterol.

Hyperlipidemia is represented by many disorders, caused by an extensive amount of lipids in the bloodstream. Patients suffering from hyperlipidemia always reveal significant increases in total cholesterol (TC), total glycerides (TG) and low-density lipoprotein cholesterol (LDL-C) levels, as well as omega-6 free fatty acids. A marked increase in omega-6 polyunsaturated acids subsequently leads to the formation of LDL/TRG [42]. Hyperglycemia is usually accompanied by an increased level of serum lipids that increases the risk factors for coronary heart disease. Many studies have shown that elevated total cholesterol (TC), total glycerides (TG), low-density lipoprotein cholesterol (LDL-C) with a concomitant reduction in high-density lipoprotein cholesterol (HDL-C) are always observed in diabetic patients [34,43]. In addition, the change in omega-6 polyunsaturated fatty acids to active metabolites is affected by the prohibition of 6-desaturase enzyme activity, triggered by insulin deficiency that consequently affects the cell membrane with its attached receptors [44]. Herein, the antihyperlipidemic effect of *S. hypoleuca* roots was evaluated after overnight fasting on the 30th day. The rats were sacrificed with anesthetic ether, by cervical dislocation and then blood was reserved for biochemical analysis. The results illustrated in Figure 3 showed a significant elevation in the TC, TG, and LDL-C levels observed in diabetic rats, estimated at 189.8%, 92.7%, and 219.8%, respectively, with a concomitant reduction in HDL-C level by 3.47% when compared to the normal group. In contrast, a significant reduction was observed in TC, TG, and LDL-C levels with concomitant elevation in HDL-C in diabetic rats treated with the root methanol extract and ethyl acetate fraction at 400 mg/kg b.wt or with standard glibenclamide. This was estimated by 39.6%, 25.6%, 63.4% and 21.8%, respectively, for root methanol extract, whereas the ethyl acetate fraction showed 46.9%, 35.3%, 65.4% and 31%, respectively; however, standard glibenclamide revealed 41.1%, 30.9%, 54.1% and 21.1%, respectively, with respect to the diabetic rats. Herein, the results depicted the reduction in TC, TG, and LDL with concomitant elevation in HDL in diabetic-treated rats in contrast to the untreated diabetic rats due to the insulin secretagogue effect of the extract, in addition to the beneficial role of HDL-C that plays an important role in the transportation of cholesterol from peripheral cells to the liver.

### 3.5. In Vivo Evaluation of Saussurea hypoleuca Roots on Liver and Kidney Markers

**Figure 4:**
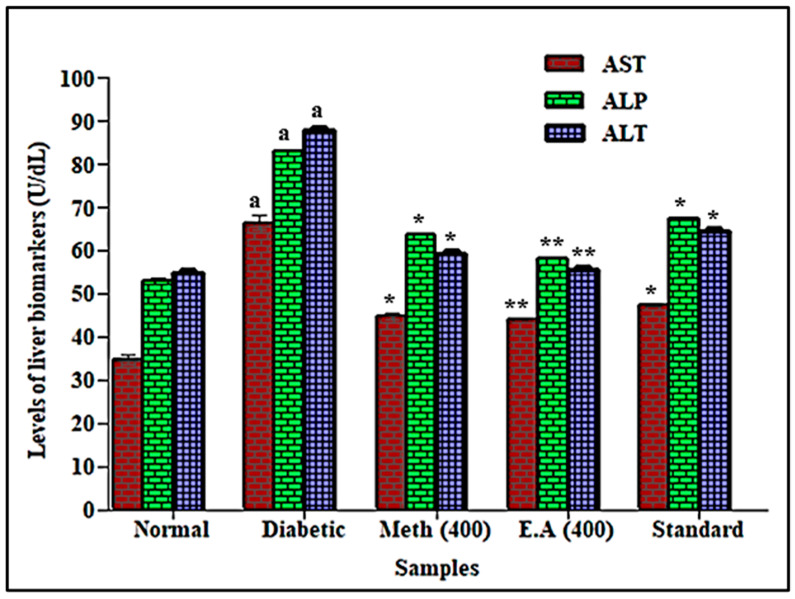
Effects of root methanol extract and ethyl acetate fraction of *S. hypoleuca* on aspartate aminotransferase (AST), alkaline phosphatase (ALP), and alanine transaminase (ALT). Values are expressed in mean ± SD, *n* = 6. Data were analyzed using Student’s *t*-test followed by one-way ANOVA. ** *p* < 0.001 and * *p* < 0.01 compared the methanol, E.A and standard with diabetic rats. ^a^ diabetic rats compared with normal rat values. AST = aspartate aminotransferase, ALP = alkaline phosphatase and ALT = alanine transaminase.

**Figure 5:**
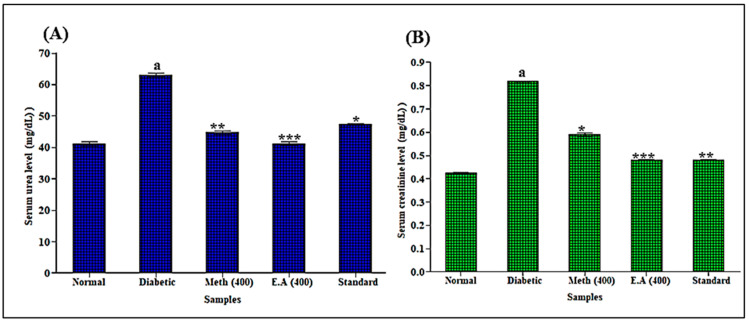
Effects of root methanol extract and ethyl acetate fraction of *S. hypoleuca* on serum urea (**A**) and serum creatinine (**B**). Values are expressed in mean ± SD, *n* = 6. Data are analyzed using Student’s t-test followed by one-way ANOVA. *** *p* < 0.0001, ** *p* < 0.001 and * *p* < 0.01 compared the methanol, E.A and standard with diabetic rats. ^a^ diabetic rats compared with normal rat values.

There are many dangerous outcomes caused by excess glucose in many body organs, such as the liver and kidney, where its reversal is crucial for good metabolic control [45]. Regarding liver markers represented by aspartate aminotransferase (AST), alkaline phosphatase (ALP), and alanine transaminase (ALT), a notable elevation was detected in diabetic rats that only received the diabetogenic agent, alloxan, causing 91.4%, 56.6%, and 60.1% elevation, respectively, with respect to the normal group. Meanwhile, a significant reduction was observed in AST, ALP, and ALT levels in diabetic rats treated with the root methanol extract and ethyl acetate fraction at 400 mg/kg b.wt or with standard glibenclamide. This was estimated as 32.3%, 23.2%, and 32.6% reductions for root methanol extract, respectively, whereas ethyl acetate fraction showed 33.8%, 30.0% and 36.7% reductions, respectively. However, standard glibenclamide revealed 28.7%, 18.9%, and 26.7% reductions, respectively, with respect to the diabetic rats (Figure 4). Similarly, a significant elevation in kidney biomarkers was exhibited by the diabetogenic agent, alloxan, in diabetic rats that received it, only causing 53.6% and 92.9% elevation in urea and creatinine levels, respectively, compared to the normal group. However, a notable decrease was observed in urea and creatinine levels in diabetic rats treated with the root methanol extract and ethyl acetate fraction at 400 mg/kg b.wt or with standard glibenclamide. This was estimated by 29.1%, and 27.9% elevation, respectively, for root methanol extract, whereas ethyl acetate fraction showed 34.7% and 41.3% elevation, respectively. However, standard glibenclamide revealed 25.0% and 41.2% elevation, respectively, with respect to the diabetic rats (Figure 5). From the illustrated results, it was obvious that in hyperglycemia, disturbance in normal metabolism of carbohydrates, lipids and proteins, along with oxidative stress, also affects hepatic and renal functions. The study also revealed the protective effects of methanol extract and ethyl acetate fraction against hepatic and kidney dysfunctions caused by diabetes. This was in line with previously reported hepatoprotective and antioxidant activities that were observed by *S. hypoleuca* roots versus paracetamol-induced hepatic damage toxicity in vivo [14]. Elevated ALP, AST, ALT, serum urea and creatinine levels in diabetic untreated rats indicated hepatic and kidney dysfunctions. It may be due to increased gluconeogenesis and ketogenesis under insulin deficiency during diabetes.

### 3.6. Histopathological Examination

**Figure 6:**
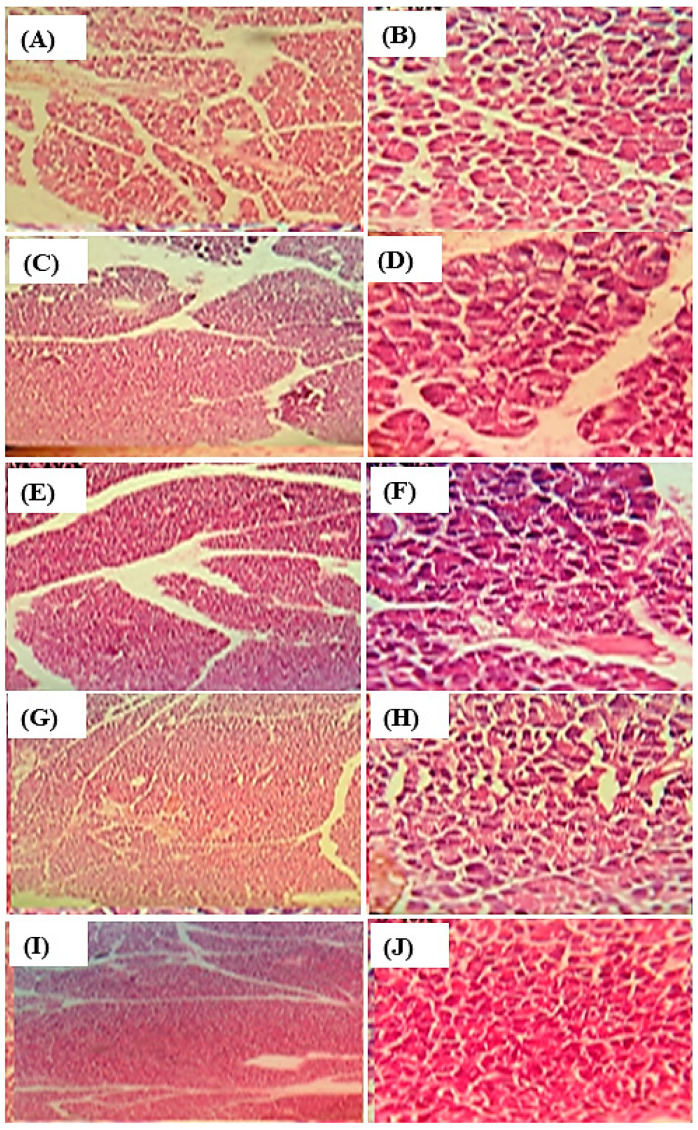
Histopathological examination of rat pancreas in normal group (10×) (**A**), 40× (**B**); diabetic group (10×) (**C**), 40× (**D**); methanol extract (10×) (**E**), 40× (**F**); and ethyl acetate fraction (10×) (**G**), 40× (**H**) and standard (10×) (**I**), 40× (**J**).

The diabetic pancreatic rats showed degenerative changes, which led to hypertrophy, inflammation, and congestion of islets of Langerhans. Meanwhile, treatment with the root methanol extract, and ethyl acetate fraction at a dose of 400 mg/kg b.wt and with glibenclamide results in full recovery. The results illustrated in Figure 6 showed that in the pancreas of normal animals, normal beta cells surrounded by dense connective tissues and normal morphology of islets Langerhans were depicted. In contrast, histological examination of the diabetic rats showed degenerations and deformities in beta cells with fatty infiltration that were partially recovered in animals treated with root methanol extract. In contrast, both the standard and the ethyl acetate fraction showed complete recovery approaching that of the normal group.

### 3.7. Molecular Docking Study

An in silico study was conducted on the identified compounds previously detected in the roots of *S. hypoleuca* by the authors [14,15,16,17] to assess their inhibitory potential versus human α-glucosidase (HAG) and ATP citrate lyase (ACL) that play a crucial role in the pathogenesis of hyperglycemia and hyperlipidemia. All the tested compounds showed certain inhibitory potential towards the examined enzymes, where decanedioic acid, myricetin, and sinapic acid revealed the highest inhibitory potential among the detected fatty acid and its derivatives, flavonoids and phenolic acid, respectively, as evidenced by their free binding energies (∆G). Regarding human alpha glucosidase, decanedioic acid, myricetin, and sinapic acid showed ∆G values of −47.68, −48.86, and −29.82 kcal/mol, respectively. In contrast, they displayed ∆G values of −40.22, −32.87, and −27.43 kcal/mol, respectively, towards ATP citrate lyase (ACL) (Table 4).

**Figure 7:**
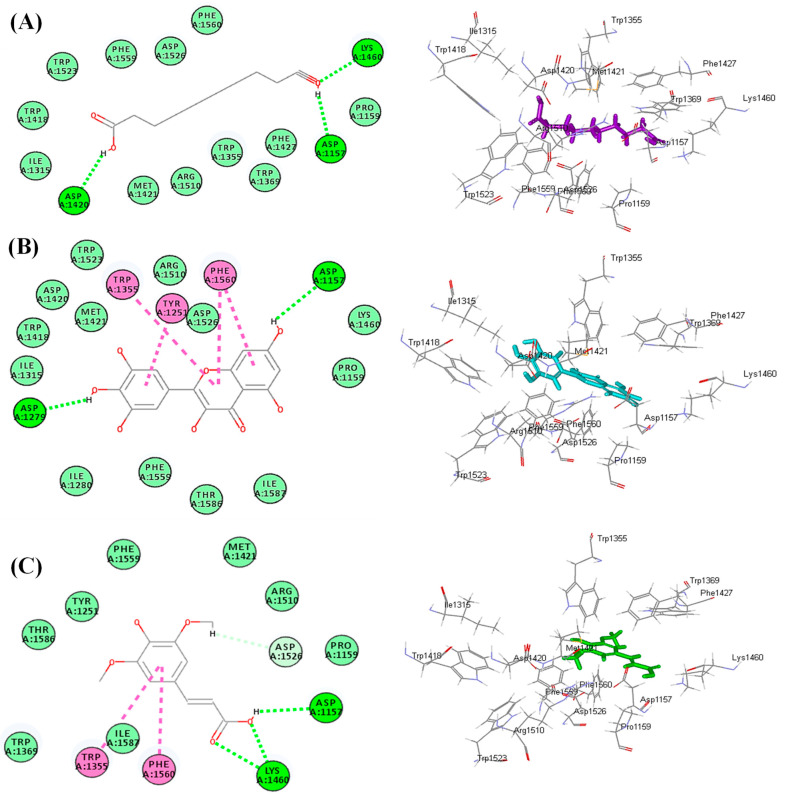
The 2D and 3D binding modes of decanedioic acid (**A**), myricetin (**B**) and sinapic acid (**C**) inside the active site of human α-glucosidase (HAG).

**Figure 8:**
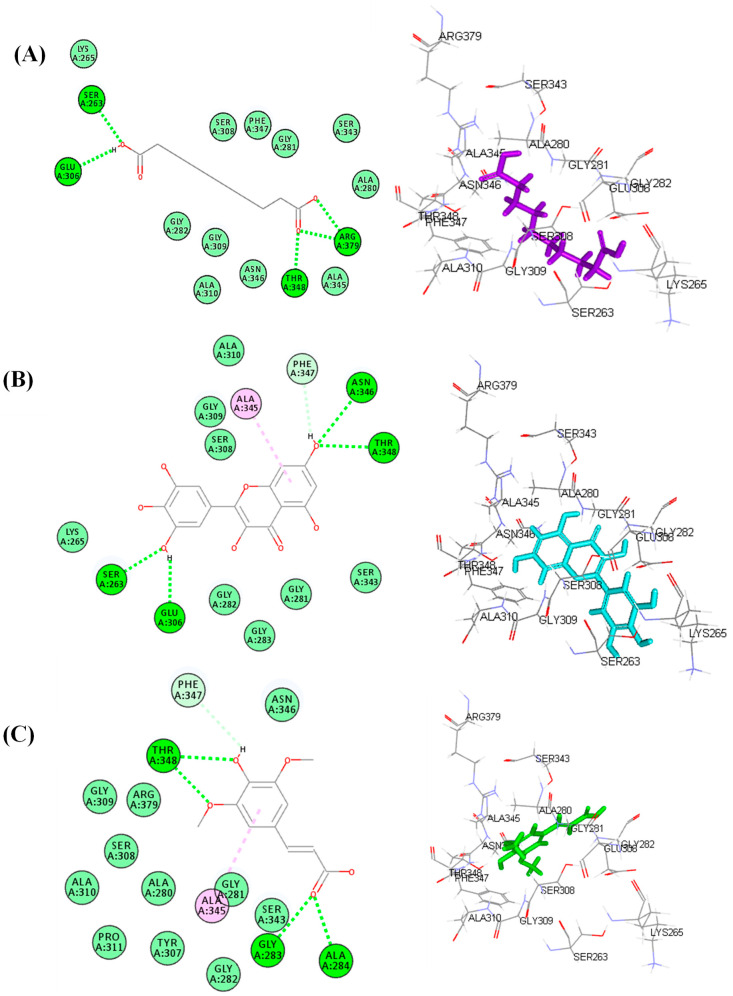
The 2D and 3D binding modes of decanedioic acid (**A**), myricetin (**B**) and sinapic acid (**C**) inside the active site of ATP citrate lyase (ACL).

The notable binding of these compounds with the active sites is probably due to the formation of multiple bonds between the functional groups of these compounds and the amino acid moieties that exist at the binding sites, in addition to the suitable size of the active compounds that fit the active pocket. Within human alpha glucosidase, decanedioic acid forms three conventional H-bonds with Lys1460, Asp1420 and Asp1157 (Figure 7A). Meanwhile, myricetin forms two conventional H-bonds with Asp1157 and Asp1279; four π-π T-shaped bonds with Trp1355, Tyr1251 and Phe1560 (Figure 7B); however, sinapic acid exerted three conventional H-bonds with Lys1460 and Asp1157; two π-π T-shaped bonds with Trp1355 and Phe1560 (Figure 7C). Concerning ATP citrate lyase, decanedioic acid forms five conventional H-bonds with Ser263, Glu306, Arg379, and Thr348 (Figure 8A). However, myricetin forms four conventional H-bonds with Ser263, Glu306, Asn346 and Thr348; one π-alkyl interaction with Ala345, in addition to one π-donor H-bond with Phe347 (Figure 8B). Meanwhile, sinapic acid forms four conventional H-bonds with Gly283, Ala284, and Thr348, in addition to one π-alkyl interaction Ala345 and one π-donor H-bond with Phe347 (Figure 8C). These results are in line with previous reports where flavonoids constitute the highly abundant and popular metabolites that showed significant antidiabetic activity [46]. They exerted their antihyperglycemic effect via a marked elevation in *β*-cell proliferation, in addition to enhancement of insulin secretion with concomitant regulation of liver glucose metabolism [47]. Furthermore, dietary phenolic acids were previously reported to reverse insulin resistance, reduce hyperglycemia, oxidative stress, hyperlipidemia and inflammation in vivo [48]. In particular, sinapic acid effectively decreases hyperglycemia in a dose dependent pattern, as previously reported. It reduced the postprandial glucose plasma with no alteration in the insulin levels, in addition to stimulating GLUT4 gene expression in the soleus muscle of STZ-diabetic rats. In addition, it stimulates hyperglycemia through phospholipase C (PLC)-protein kinase C (PKC) (PLC-PKC) signals to stimulate glucose utilization in diabetic rats [49].

## 4. Conclusions

In the foregoing study, the in vivo antihyperglycemic and antihyperlipidemic activity evaluation of *Saussurea hypoleuca* roots in alloxan-induced diabetes in a rat model revealed that both the root methanol extract and ethyl acetate fraction exhibited potent activity. This was evidenced by the significant amelioration in FBG level and HbA_1c_ as antihyperglycemic activity descriptors that were further consolidated by the histopathological examination of the animals’ pancreatic sections. Meanwhile, they effectively reduced TC, TG and LDL-C with concomitant elevation in HDL-C, manifesting their pronounced antihyperlipidemic potential. This was accompanied by significant effects as hepatoprotective and nephroprotective agents, as reflected in the normalization of AST, ALT, ALP, urea, and creatinine levels as liver and kidney biomarkers that reflect their healthy state. This is mainly attributed to the richness of *S. hypoleuca* roots with different secondary metabolites that belong to different classes, particularly fatty acids and their derivatives, flavonoids, as well as phenolic acid that works synergistically to produce the observed activity. This was further confirmed by an in silico molecular modelling study, where all of the tested compounds showed certain inhibitory potential towards human α-glucosidase (HAG) and ATP citrate lyase (ACL) that play a crucial role in the pathogenesis of hyperglycemia and hyperlipidemia. Decanedioic acid, myricetin, and sinapic acid revealed the highest inhibitory potential among the detected fatty acids and their derivatives, flavonoids, and phenolic acid, respectively. It was concluded that the beneficial effects of *S. hypoleuca* roots could help in the treatment of hyperglycemia, hyperlipidemia, and hepatic and kidney dysfunction. Thus, *S. hypoleuca* could be used as a potential source of therapeutically active compounds, which would be helpful for the discovery of clinically effective and safe drugs in managing diabetes.

## Figures and Tables

**Table 1:** Effects of *S. hypoleuca* root methanol extract and its successive fractions on fasting blood glucose (FBG) levels in the short-term study.

Groups	0 h	1 h	2 h	3 h	4 h	5 h	6 h
Normal	75.9 ± 7.0	77.3 ± 7.6	76.6 ± 6.0	79.2 ± 4.8	76.1 ± 6.6	78.4 ± 2.8	77.5 ± 4.9
Diabetic	309.2 ± 11.5 †	305.3 ± 10.3	333.9 ± 14.7	347.8 ± 18.9	319.9 ± 16.6	326.2 ± 15.3	357.0 ± 16.4
Methanol	300.9 ± 16.4 †	260.8 ± 16.3	246.9 ± 8.61	240.1 ± 9.6	232.7 ± 8.0 *	229.6 ± 9.4 *	204.5 ± 8.6 **
*n*-Hexane	307.9 ± 12.1 †	300.2 ± 17.7	308.2 ± 15.2	311.5 ± 13.7	310.4 ± 13.5	315.5 ± 18.1	323.7 ± 13.0
Chloroform	299.0 ± 15.9 †	295.7 ± 15.3	304.5 ± 15.6	314.3 ± 13.9	304.7 ± 12.7	317.0 ± 11.2	322.2 ± 12.3
Ethyl acetate	334.0 ± 14.6 †	263.8 ± 12.7	223.8 ± 12.1	162 ± 10.9 **	119.0 ± 8.8 **	92.3 ± 10.8 **	79.2 ± 4.8 **
*n*-Butanol	294.4 ± 16.7 †	290.3 ± 15.4	298.7 ± 16.7	300.8 ± 14.0	303.7 ± 15.6	307.0 ± 13.9	311.2 ± 16.3
Aqueous	298.0 ± 13.9 †	293.7 ± 13.2	300.9 ± 16.4	309.2 ± 11.5	307.0 ± 20.1	313.7 ± 10.4	320.5 ± 17.0

Results are summarized as mean ± standard deviation (SD); *n* = 6. Data analysis was performed using Student’s *t*-test followed by one-way ANOVA. ** *p* < 0.001, * *p* < 0.01 compared with initial levels of FBG (0 h) of group, † *p* < 0.001 compared with initial level of FBG (0 h) of normal group.

**Table 2:** Effects of root methanol extract and ethyl acetate fraction of *S. hypoleuca* on FBG levels administered at different doses.

Groups	0 h	1 h	2 h	3 h	4 h	5 h	6 h
Normal	76.6 ± 7.2	78.9 ± 8.4	76.4 ± 5.8	75.9 ± 6.6	73.9 ± 7.4	78.1 ± 5.2	70.7 ± 4.1
Diabetic	334.12 ± 11.4 †	333.7 ± 12.4	329.7 ± 13.2	355.2 ± 11.6	343.23 ± 11.8	353.2 ± 5.4	351. ± 12.3
Meth (200)	300.9 ± 16.4 †	260.8 ± 16.3	246.9 ± 8.6 *	240.1 ± 9.6 *	232.7 ± 8.0 *	229.6 ± 9.4 *	204.5 ± 8.6 *
Meth (400)	308.6 ± 13.9 †	276.3 ± 12.7	215.5 ± 14.9 *	161.6 ± 12.1 **	116.8 ± 8.7 **	97.4 ± 8.0 **	81.4 ± 10.0 **
E.A (200)	330.1 ± 12.3 †	260.6 ± 11.3	220.7 ± 10.5 *	165.4 ± 8.1 **	117.7 ± 9.2 **	88.9 ± 7.8 **	79.3 ± 5.4 **
E.A (400)	313.1 ± 11.3 †	269.3 ± 10.8	211.0 ± 7.9 *	165.2 ± 19.7 **	136.2 ± 10.0 **	113.9 ± 8.1 **	70.9 ± 8.6 **
Standard	322.6 ± 6.6 †	291.9 ± 7.6	271.6 ± 8.1	248.9 ± 11.1 *	223.6 ± 6.8 *	202.9 ± 8.7 **	226.6 ± 15.8 *

Results are summarized as mean ± SD, *n* = 6. Data analysis performed using Student’s t-test followed by one-way ANOVA. ** *p* < 0.001, * *p* < 0.01 compared with initial levels of FBG (0 h) of group, † *p* < 0.001 compared with initial level of FBG (0 h) of normal groups. Meth (200) and Meth (400); *S. hypoleuca* root methanol extract at a dose of 200 and 400 mg/kg b.wt, respectively; E.A (200) and E.A (400); *S. hypoleuca* root ethyl acetate fraction at a dose of 200 and 400 mg/kg b.wt, respectively; standard, glibenclamide at a dose of 10 mg/kg b.wt.

**Table 3:** Effects of root methanol extract and ethyl acetate fraction of *S. hypoleuca* on FBG levels and animal body weights (long-term study).

Groups	1st d	10th d	20th d	30th d	Weight (g)
Normal	82.1 ± 5.3 ^a^	89.4 ± 6.2 ^a^	87.1 ± 5.3 ^a^	83.8 ± 4.9 ^a^	218.2 ± 5.3 ^a^^b^
Diabetic	350.9 ± 7.6 ^b^	423.2 ± 5.3 ^c^	431.0 ± 9.1 ^d^	444.6 ± 5.9 ^e^	170.7 ± 8.0 ^c^
Meth (400)	310.1 ± 7.8 ^b^	179.2 ± 7.6 ^a^	104.5 ± 6.6 ^a^	102.2 ± 5.3 ^a^	216.8 ± 8.9 ^a^^b^
E.A (400)	292.7 ± 15.7 ^b^	186.9 ± 4.2 ^a^	105.1 ± 6.0 ^a^	101.3 ± 6.1 ^a^	216 ± 5.1 ^a^^b^
Standard	309.4 ± 7.8 ^b^	276.6 ± 4.7 ^b^	242.3 ± 8.7 ^b^	207.5 ± 6.7 ^b^	216.8 ± 4.1 ^a^^b^

Values are expressed as mean ± SD, *n* = 6, all values that have common superscript letters differ significantly at *p* < 0.01 (DMRT); Meth (400); *S. hypoleuca* root methanol extract at a dose of 400 mg/kg b.wt; E.A (400); *S. hypoleuca* root ethyl acetate fraction at a dose of 400 mg/kg b.wt; standard, glibenclamide at a dose of 10 mg/kg b.wt, DMRT = Duncan’s multiple range test.

**Table 4:** In silico studies expressed as free binding energies (kcal/mol) for the predominating identified compounds present in *Saussurea hypoleuca* roots within the active site of human α-glucosidase (HAG) and ATP citrate lyase (ACL).

Compounds	Human α-Glucosidase (HAG)	ATP Citrate Lyase (ACL)
Decanedioic acid	−47.68	−40.22
Dioctyl ether	−37.33	−29.99
Hexadecanoic acid	−46.42	−34.84
Isopropyl myristate	−43.86	−34.15
Oleic acid	−29.48	−23.86
Tetracosapentaene	−32.76	−15.70
Kaempferol	−37.05	−22.29
Luteolin	−43.28	−30.32
Myricetin	−48.86	−32.87
Quercetin	−43.90	−26.51
Sinapic acid	−29.82	−27.43
Caffeic acid	−28.42	−23.22

## Data Availability

Data are available in the manuscript.
